# MicroRNA-361-5p acts as a biomarker for carotid artery stenosis and promotes vascular smooth muscle cell proliferation and migration

**DOI:** 10.1186/s12920-023-01563-2

**Published:** 2023-06-16

**Authors:** Fei Wang, Yumei An, Huihui Hao

**Affiliations:** 1 Department of Neurosurgery, Affiliated Hospital of Weifang Medical University, No. 2428 Yuhe Road, Kuiwen District, 261035 Weifang, Shandong China; 2 Department of Image Center, Affiliated Hospital of Weifang Medical University, 261035 Weifang, China; 3 Department of Pharmacy, Affiliated Hospital of Weifang Medical University, 261035 Weifang, China

**Keywords:** Carotid artery stenosis, MicroRNA-361-5p, ROC, VSMCs

## Abstract

**Background:**

Vascular smooth muscle cells (VSMCs) dysfunction participates in carotid artery stenosis (CAS). The study aimed to examine the expression pattern of miR-361-5p in CAS patients, and explore its role in VSMCs proliferation and migration.

**Methods:**

qRT-PCR was performed for the detection of miR-361-5p in serum samples of 150 CAS cases and 150 healthy people. Multiple logistic regression analysis and receiver operating characteristic (ROC) curve was accomplished to detect diagnostic value via SPSS 21.0 statistical software. Cell function of VSMCs was evaluated. Target association was predicted through bioinformatic analysis and confirmed via luciferase activity.

**Results:**

Serum miR-361-5p was enhanced in CAS cases and was positively correlated with CAS degree. Logistic regression analysis determined the independent influence of miR-361-5p in CAS, and ROC curve demonstrated its diagnostic value with AUC of 0.892. miR-361-5p promoted VSMCs proliferation and migration, but the influence was counteracted by TIMP4.

**Conclusions:**

MiR-361-5p is a promising biomarker for CAS, and can be used as a potential target for early diagnosis and treatment of CAS. MiR-361-5p can promote VSMCs proliferation and migration via targeting TIMP4.

## Background

Ischemic stroke (IS) refers to the loss of local blood supply to the brain tissue caused by various reasons, resulting in ischemic hypoxic lesion necrosis of the brain tissue [1]. The clinical manifestation is the loss of corresponding neural function [2]. Carotid artery is one of the main blood supply vessels in the brain, and carotid artery stenosis (CAS) is the main inducement of cerebral infarction [3]. Patients with severe carotid artery stenosis still have a higher incidence of cerebral ischemia events even after effective treatment [4]. Therefore, it is of great significance to explore the biological factors and markers related to carotid artery stenosis for the early diagnosis, timely treatment and prognosis of cerebral infarction patients. According to statistics, about half of IS is caused by CAS, which is related to carotid atherosclerosis [5]. The pathogenesis of carotid atherosclerosis is a complicated process, in which vascular smooth muscle cells (VSMCs) and endothelial cell dysfunction and macrophage plaque infiltration play an important role, especially VSMCs [6]. VSMCs have close association with the plaque growth at an early stage and the stability at a later stage [7]. It suggests that VSMCs may be an effective therapeutic target for CAS [8].

MicroRNAs (miRNAs) which are small noncoding RNAs, play a key role in the epigenetic regulation of gene expression and control many metabolic and physiological processes associated with health and disease [9, 10]. It is well known that the small non-coding RNAs (including miRNAs) are known to be the gene post-transcriptional regulators in the cell proliferation, apoptosis, migration, and invasion pathways [11]. MiRNA plays a key role in the pathophysiology of IS by mediating angiogenesis, apoptosis and oxidative stress [12]. Based on the reported studies, the important role of miR-361-5p in cardiovascular and cerebrovascular diseases has been widely elucidated. In oxygen glucose deprivation/reoxygenation (OGD/R) induced cerebral ischemia and reperfusion (I/R) injury cell model, upregulated of miR-361-5p is tested, which was related to the cell apoptosis [13]. In IS mice models, the overexpression of miR-361-5p is also reported to brain infarct volume [14]. Notably, Dolz et al. also determine the upregulation of miR-361-5p in the serum of patients with asymptomatic CAS via Affymetrix microarrays [15]. Furthermore, a close relationship of miR-361-5p with atherosclerosis has been presented by Ling et al. in both in vivo and in vitro experiments [16]. Thereby, the role and underlying mechanism of miR-361-5p in CAS caught our attention.

In the current study, miR-361-5p expression pattern and diagnostic value was evaluated in CAS patients. In vitro, role and potential mechanism of miR-361-5p in VSMCs proliferation and migration was also investigated.

## Methods

### Study subjects

150 cases with CAS were enrolled in the current study, who were diagnosed based on carotid artery ultrasound and angiography in Affiliated Hospital of Weifang Medical University. The imaging results were diagnosed by two doctors. All enrolled patients had a degree of CAS > 50%, and divided into moderate group (50–69% stenosis) and severe group based on the CAS degree (≥ 70% stenosis) [17, 18]. Another 150 age and gender matched healthy individuals were collected as the control groups, and they underwent carotid artery ultrasound to avoid the appearance of CAS (carotid artery stenosis < 20%). The exclusion criteria were as follows: (1) Age below 18 years: (2) malignant tumor; (3) Acute or chronic infection; (4) angiography contraindicated; (5) Recent history of surgical trauma; (6) Previous history of carotid artery surgery; (7) systemic immune diseases; (8) Use of anti-inflammatory drugs.

The procedures used in this study adhere to the tenets of the Declaration of Helsinki. And this study was reviewed and approved by the Ethics Committee of Affiliated Hospital of Weifang Medical University. All subjects were informed of the procedure of the study design and signed informed consent.

### Clinical laboratory data collection

Basic information of the enrolled study subjects was collected after admission, including age, weigh, height, sex, systolic blood pressure (SBP), diastolic blood pressure (DBP), smoke, heart rate (HR). And the pulse pressure (PP) and body mass index (BMI) were calculated based on the formula [PP = SBP (mmHg) - DBP (mmHg)], BMI = weight (kg)/height (m^2^) ].

5 ml fasting peripheral venous blood was gained from each people. After centrifugation at 3000 rpm for 15 min, the serum samples were collected for laboratory data detection, including fasting blood-glucose (FBG), mean platelet volume (MPV), triglyceride (TG), high-density lipoprotein cholesterol (HDL-C), low-density lipoprotein cholesterol (LDL-C). The commercially available solid phase enzymelinked immunosorbent assay (ELISA) kits (R&D Systems, Abingdon, UK) were used for the measurement of TIMP-4 protein levels in the serum samples.

### Cell culture and transfection

Human VSMCs were provided by Sciencell (San Diego, CA, USA), and cultured in SMC medium (Sciencell, CA, USA) at 37 ℃ and 5% CO_2_. In the process of cell culture, the aseptic operation principle was strictly followed, and the medium was changed every 2–3 days. When the cell growth and fusion were over 90%, 0.25% trypsin (without EDTA) was used for digestion and passage.

Cell transfection was accomplished to control miR-361-5p levels in VSMCs, and sequences of miR-361-5p mimic, the negative control od miR-361-5p mimic (mimic NC), miR-361-5p inhibitor, and its negative control (inhibitor NC) were synthesized by Ruibo biological technology co. (Guangzhou, China). Cell transfection was detailed described in Table 1. The small interfering RNA of TIMP4 (si-TIMP4) was used for the downregulation of TIMP4, which was also synthesized by Ruibo biological technology co. (Guangzhou, China). Lipofectamine 2000 was applied to the cell transfection in line with the specification. 48 h after transfection, cells from each group were collected for follow-up experiments.

**Table 1:** Conditions of cell grouping

Groups	Cell transfection
Mock	Normal culture
MiR-361-5p mimic	50 nM miR-361-5p mimic
Mimic-NC	50 nM mimic-NC
MiR-361-5p inhibitor	100 nM miR-361-5p inhibitor
Inhibitor NC	100 nM inhibitor NC

### RT-qPCR assay

Total RNA was separated by Trizol reagent, miRNA was reverse-transcribed into cDNA using miRcute Plus miRNA First-Strand cDNA Kit, and then miRcute Plus miRNA qPCR Kit (SYBR Green) was used for RT-qPCR. FastKing gDNA Dispelling RT SuperMix and SuperReal PreMix Plus were used for reverse-transcription and RT-qPCR respectively. All used kits were purchased from Tiangen Biotech Co. (Beijing, China). RT-qPCR was proceeded on the ABI 7500 real-time PCR system under the following reaction conditions: predenaturation at 95℃ for 1 min,95 ℃ for 15 s,60℃ for 15 s, a total of 40 cycles. U6 and GAPDH were identified as the standardized internal reference for miR-590-5p and TIMP4. Relative mRNA levels were calculated by 2^−ΔΔCt^ method.

### Cell counting kit-8 (CCK-8) assay

The 96-well plates were used for the cell incubation of VSMCs with the density of 5 × 10^4^. Cells were cultured for 3 days continuously and 10 µL CCK-8 was added at 0 h, 24 h, 48 and 72 h, respectively. After incubating at 37 ℃ and 5% CO_2_ for 3 h, the absorbance values of cells at 450 nm were measured.

### Transwell assay

The cells were incubated in an incubator containing 5% CO_2_ at 37 ℃ for 24 h, then the medium was discarded. Then cells were cleaned with PBS for 3 times. Gently wipe the cells on the upper wall of the upper chamber with a cotton swab, and then fix the cells using 4% paraformaldehyde for 20 min. After cleaning with PBS for 3 times to thoroughly remove formaldehyde, the chamber was immersed in hematoxylin solution for 20 min. After 3 times of cleaning with PBS, the cells were counted under a microscope, and the cell numer in the lower chamber was calculated through Image pro Plus software.

### Luciferase reporter assay

The pmirGLO vectors containing the wide type (WT-TIMP4) or mutant type (MUT-TIMP4) fragments of TIMP4 3’-UTR containing the predicted miR-361-5p binding sites were constructed. Then the reporter vector and miR-361-5p mimics, miR-361-5p inhibitor, mimic NC or inhibitor NC were co-transfected into VSMCs via Lipofectamine 2000. The luciferase activity of the cells was monitored after 48 h of culture.

### Statistics process

The statics process was accomplished via SPSS 21.0 statistical software. The continuous variable were shown as mean and standard deviation (SD), and the difference comparison was proceed using one-way analysis of variance (ANOVA). Counting data were compared by chi-square test. The difference was identified to be statistically significant when *P* value less than 0.05.

## Results

### General data of the two study groups

In the CAS group, there were 79 males and 71 females, the mean age was 55.21 ± 7.78 years old (Table 2). Another 82 males and 68 females formed the control group, with the mean age of 54.55 ± 8.50 years old. The age, gender and BMI of CAS group did not differ from the control group (*P* > 0.05). More smokers were found in CAS group than the control group (*P* < 0.05). Cases in the CAS group owned high values of FBG, LDL-C, SBP, DBP and PP, and low levels of LDL-C compared with the control group (*P* < 0.05). But other laboratory data indicated no obvious difference between the two groups, including MPV, TG and HR (*P* > 0.05).

**Table 2:** Comparison of general data between the two study groups

Items	Control group (n = 150)	CAS group (n = 150)	*P* value
Age, year	54.55 ± 8.50	55.21 ± 7.78	0.484
Sex, male/female	82/68	79/71	0.728
BMI, kg/m^2^	24.78 ± 3.19	24.93 ± 3.09	0.681
**Smoker**, n (%)	66/84	87/63	**0.011**
**FBG**, mmol/L	5.81 ± 0.51	6.03 ± 0.55	**< 0.001**
MPV	10.18 ± 0.98	10.25 ± 1.09	0.584
TG, mmol/L	1.56 ± 0.44	1.66 ± 0.62	0.108
**HDL-C**, mmol/L	1.24 ± 0.51	1.07 ± 0.50	**0.003**
**LDL-C**, mmol/L	2.05 ± 0.64	2.23 ± 0.79	**0.031**
HR	76.22 ± 10.94	77.17 ± 9.97	0.431
**SBP**	126.35 ± 14.00	147.13 ± 21.39	**< 0.001**
**DBP**	82.58 ± 11.06	89.43 ± 13.71	**< 0.001**
**PP**	43.77 ± 17.08	57.70 ± 26.42	**< 0.001**

Note: FBG, fasting blood-glucose; MPV, mean platelet volume; TG, triglyceride; HDL-C, high-density lipoprotein cholesterol; LDL-C, low-density lipoprotein cholesterol; HR, heart rate; SBP, Systolic Blood Pressure; DBP, Diastolic Blood Pressure; PP, Pulse pressure

### Expression pattern of mir-361-5p in CAS cases

**Fig. 1:**
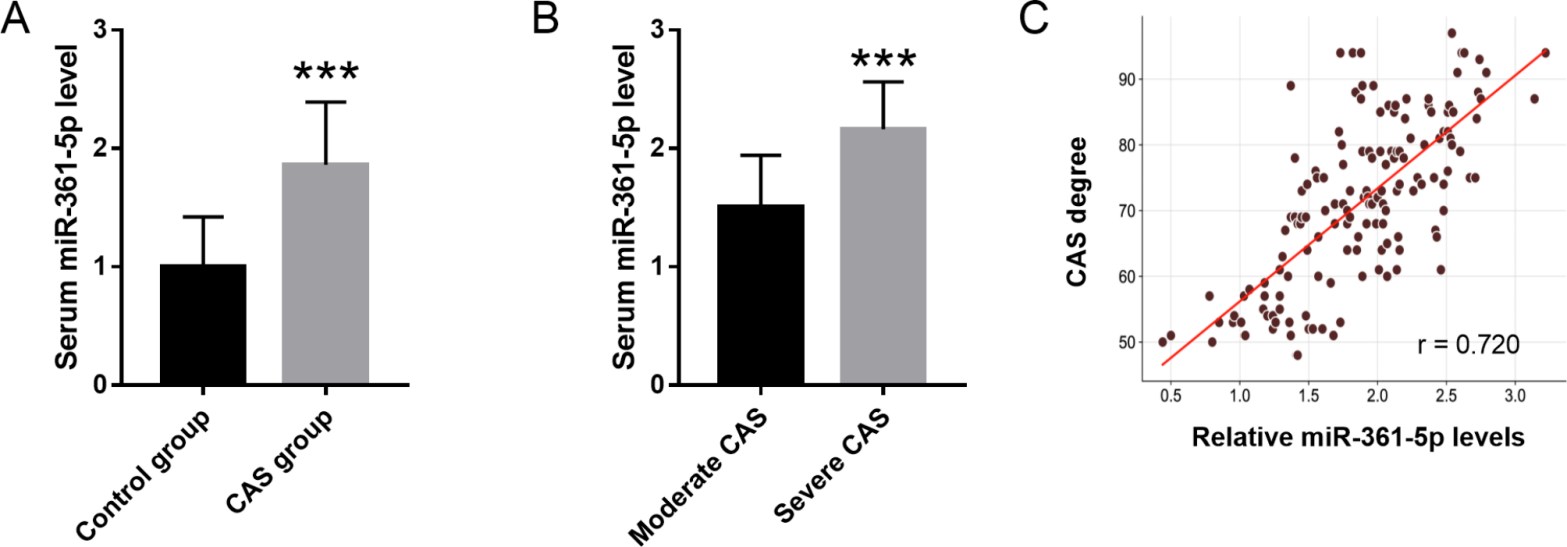
Expression pattern of miR-361-5p in CAS cases. **A**. Elevated level of miR-361-5p was detected in the serum of CAS cases in comparison with the control group. **B**. High values of serum miR-361-5p was determined in severe CAS group. **C**. Correlation of serum miR-361-5p with the CAS degree score. *** *P* < 0.001

As shown in Fig. 1A, qRT-PCR assay pointed out the elevated value of miR-361-5p in the serum of CAS cases in contrast to the control group (*P* < 0.01). According to the CAS degree, 150 CAS patients were splitted into moderate group (n = 67) and severe group (n = 83), and high standard serum miR-361-5p was determined in severe CAS group (Fig. 1B, P < 0.01). Furthermore, Pearson’s correlation analysis results suggested that elevated serum miR-361-5p levels were positively correlated with the CAS degree score (r = 0.720, Fig. 1C).

### Clinical value of mir-361-5p for CAS

**Fig. 2:**
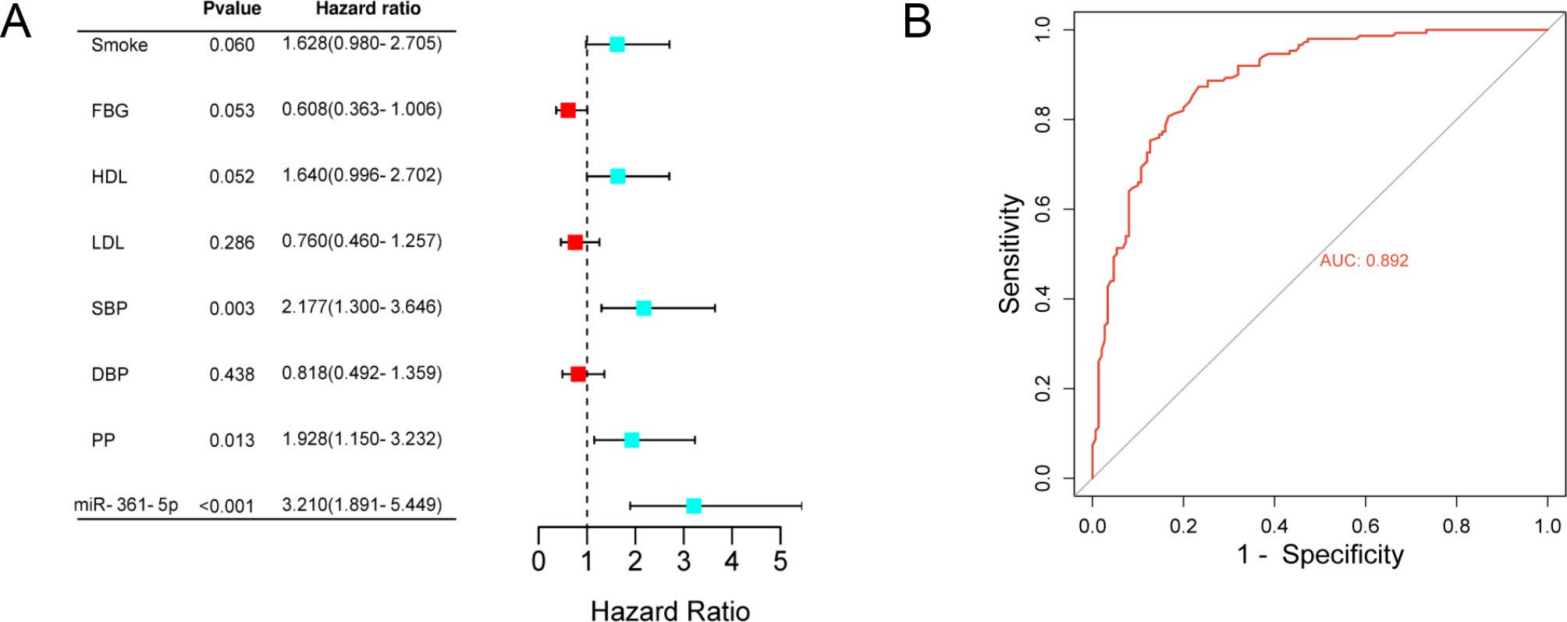
Clinical value of miR-361-5p for CAS. **A**. Multiple logistic regression analysis results of clinical factors related to CAS and the forest plot. **B**. ROC curve of serum miR-361-5p in differentiating CAS from healthy people

Based on the comparison results in Table 2, the significantly different factors were intaken into the multiple logistic regression model combined with serum miR-361-5p to analyze their independent clinical value for CAS. As illustrated in Fig. 2, miR-361-5p (HR = 3.210, 95% CI = 1.891–5.449, *P* < 0.01), SBP (HR = 2.177, 95% CI = 1.300-3.646, *P* < 0.01) and PP (HR = 1.928, 95% CI = 1.150–3.232, *P* < 0.05) were independent predictors for the onset of CAS. Furthermore, the receiver operating characteristic (ROC) curve was drawn according to the serum miR-361-5p levels in CAS and control groups, in order to evaluate its diagnostic value for CAS. As pictured in Fig. 2B, serum miR-361-5p can differentiate CAS cases from healthy people with the area under the curve (AUC) of 0.892. The diagnostic sensitivity and specificity were 83.3% and 80.7%, respectively.

### Mir-361-5p promotes VSMCs proliferation and migration

**Fig. 3:**
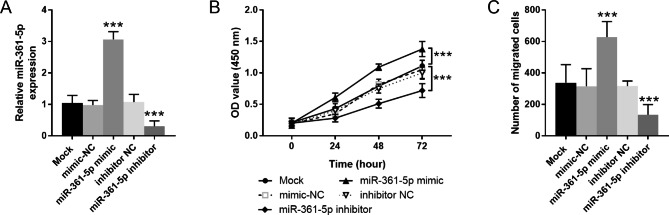
MiR-361-5p promotes VSMCs proliferation and migration. **A**. MiR-361-5p levels in cells after transfected with miR-361-5p mimic or inhibitor. **B**. Cell proliferation of cells after transfected with miR-361-5p mimic or inhibitor. **C**. Cell migration of cells after transfected with miR-361-5p mimic or inhibitor. *** *P* < 0.001 vs. control group

Figure 3 A reflected the transfection efficiency, the results determined that miR-361-5p mimic transfection bring about the elevation of miR-361-5p in VSMCs, while miR-361-5p inhibitor transfection had the opposite effect. The CCK-8 assay determined that miR-361-5p overexpression facilitated VSMCs proliferation, but miR-361-5p inhibitor played an inhibitory impact on cell proliferation (Fig. 3B). The Transwell assay results also demonstrated the enhancement role of miR-361-5p in VSMCs migration, and miR-361-5p inhibitor suppressed the cell migration (Fig. 3C).

### TIMP4 serves as the target gene of miR-361-5p

**Fig. 4:**
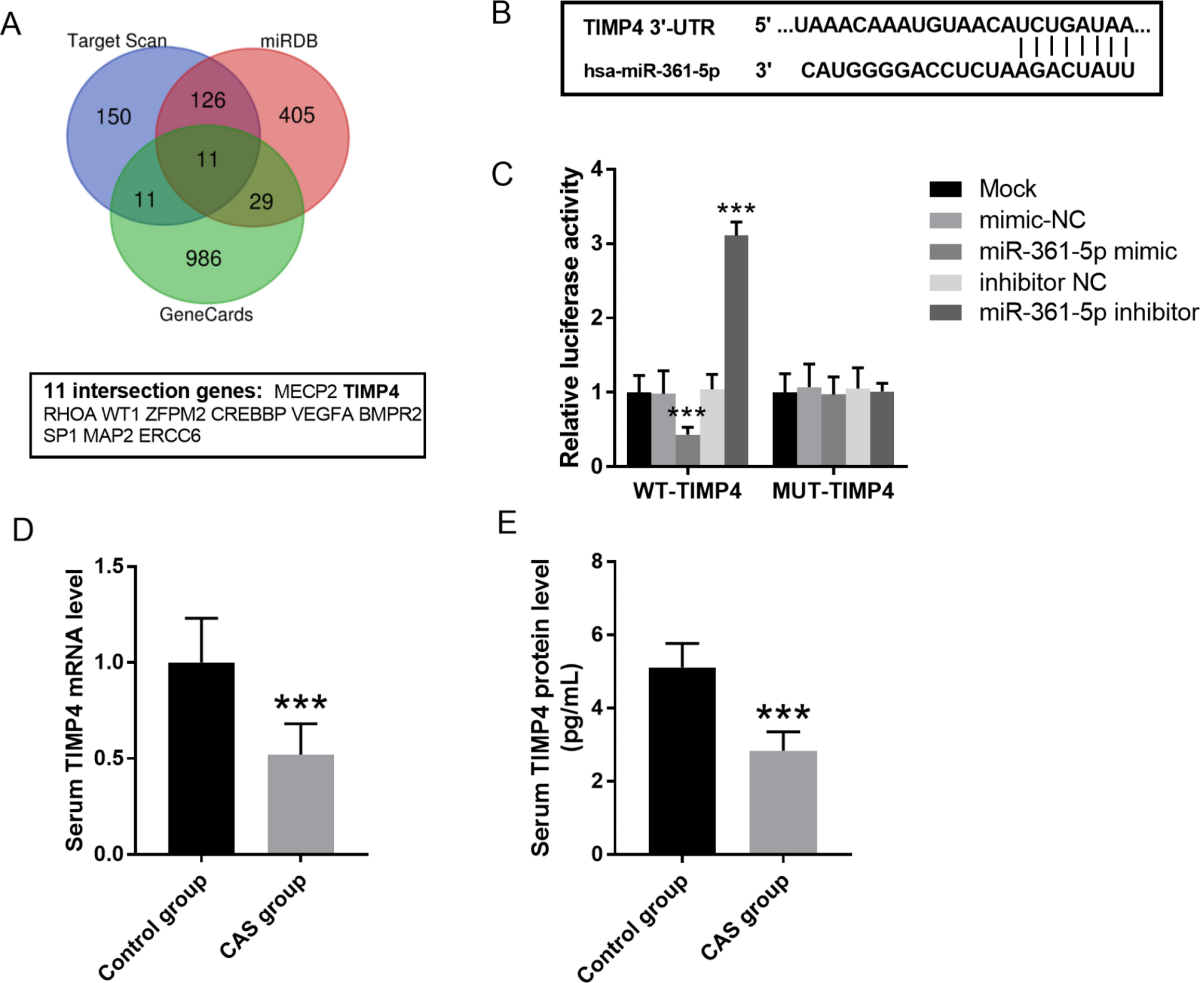
TIMP4 serves as the target gene of miR-361-5p. **A**. The Venn diagram displayed the intersection genes based on the data from TargetScan, miRDA and GeneCards. B. The binding sites between miR-361-5p and TIMP4 was predicted by TargetScan. **C**. Cell luciferase activity of cells after transfected with miR-361-5p mimic or inhibitor. *** *P* < 0.001 vs. mock group. **D**. Decreased TIMP4 mRNA levels in the serum of CAS cases. **E**. Decreased TIMP4 protein levels in the serum of CAS cases. *** *P* < 0.001

The Venn diagram displayed the intersection genes based on the data from TargetScan, miRDA and GeneCards, which was drawn using the jvenn online tool. As shown in Fig. 4A, a total of 11 intersection genes were identified as the candidate target gene of miR-361-5p which related to CAS. Among them, TIMP4 was selected for the further research, because its close relationship with CAS and atherosclerosis as previously reported. As observed in Fig. 4B, the binding site between miR-361-5p and TIMP4 was predicted by TargetScan. Luciferase reporter assay was accomplished for the validation of target gene. It can be seen from Fig. 4C that, for WT-TIMP4 transfected VSMCs, miR-361-5p mimic transfection decreased the luciferase activity, but miR-361-5p inhibitor significantly strengthened the cells’ luciferase activity (*P* < 0.01). Additionally, decreased TIMP4 mRNA and protein levels were also detected in the serum of CAS cases (Fig. 4D-E).

### TIMP4 knockdown counteracts the role of mir-361-5p in VSMCs

**Fig. 5:**
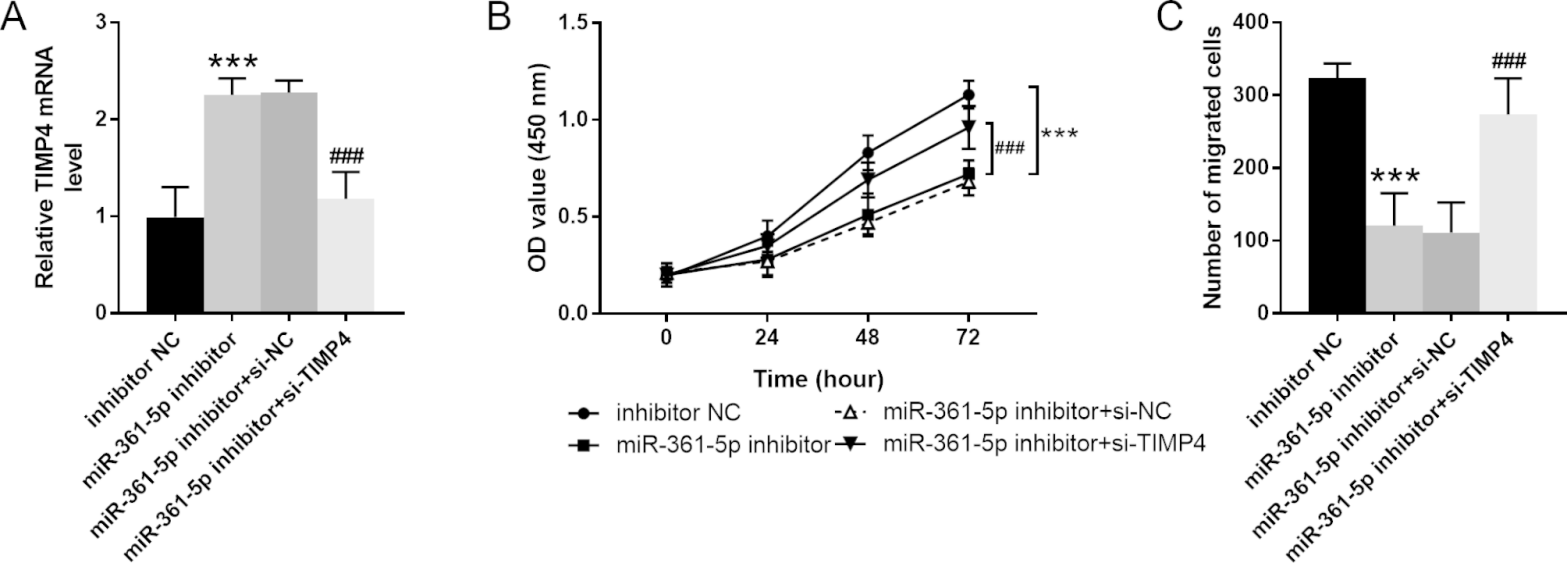
TIMP4 knockdown counteracts the role of miR-361-5p in VSMCs. **A**. TIMP4 mRNA levels in cells after transfected with miR-361-5p inhibitor or si-TIMP4. **B**. Cell proliferation of cells after transfected with miR-361-5p inhibitor or si-TIMP4. **C**. Cell migration of cells after transfected with miR-361-5p inhibitor or si-TIMP4. *** *P* < 0.001 vs. inhibitor NC group; ### *P* < 0.001 vs. miR-361-5p inhibitor group

As observed from Fig. 5A, miR-361-5p inhibitor transfection accompanied with the upregulation of TIMP4 mRNA, which was restrained by si-TIMP4. The CCK-8 and Transwell assay determined that the suppression of both cell proliferation and migration caused by miR-361-5p was counteracted by TIMP4 knockdown (Fig. 5B-C).

## Discussion

CAS is a progressive atherosclerotic disease [19]. Atherosclerosis-related mortality remains the most common cause of death worldwide [20]. Atherosclerotic carotid plaque rupture may lead to thromboembolization, causing transient ischemic attack or ischemic stroke [21, 22]. Due to the increasing annual incidence rate of disability and mortality in patients with CAS, the need for an appropriate diagnostic tool has become a crucial urgent issue [22]. An increase in biomarkers and protein levels in response to CAS can be used as a predictive biomarker with different sensitivities and specificities, such as such as miR-1, miR-221-3p and miR-19a [23, 24]. In the current study, qRT-PCR assay pointed out the elevated level of miR-361-5p in the serum of CAS cases, the findings were consistent with the previous evidence reported by Dolz et al. [15]. These findings support our speculation about the important role of miR-361-5p in CAS.

As previously reported, miR-361-5p is an atherosclerosis-related miRNA [16]. In a study about the role of Long non-coding RNA MDRL in atherosclerosis, a close relationship is presented between miR-361-5p and MDRL, which is involved in the NLRP3 inflammasome activation and apoptosis in VSMCs during atherogenesis [16]. Atherosclerosis is the main inducement of most CAS and is connected with the progression of CAS [25]. Considering the crucial role of miR-361-5p in atherosclerosis, its clinical values in CAS was evaluated. As Pearson’s correlation analysis results suggested, elevated serum miR-361-5p levels were positively correlated with the CAS degree score. And CAS cases with severe CAS owned high levels of miR-361-5p. miR-361-5p may promote carotid atherosclerosis and plaque formation, further lead to the worsening of carotid artery stenosis [16]. Clinically, there are many important risk factors for CAS, such as hypertension, diabetes, hyperlipidemia, smoking, etc. [26]. In the present study, the basic clinical information of CAS cases and controls were compared. It was found that more smokers were found in CAS group than in the control group. In addition, CAS cases owned significantly high levels of FBG, LDL-C, ABP, DBP, and low levels HDL-C and PP. Furthermore, these significantly different factors were included in the logistic regression model combined with serum miR-361-5p to analyze their independent clinical value. The results showed that miR-361-5p, SBP and PP were independence predictors for the onset of CAS. And serum miR-361-5p was demonstrated a high diagnostic value for CAS.

The abnormal vascular smooth muscle cells (VSMC) proliferation and migration are the key factors in the progression of atherosclerotic plaque and CAS [10, 27–29]. There are abundant reports about the effects of miRNAs on the proliferation and migration of VSMCs [30]. In light of the abnormal expression of miR-361-5p in clinical serum samples, its role in VSMC function was explored. The rescue experiments illustrated that miR-361-5p overexpression promoted VSMC proliferation and migration, while miR-361-5p inhibitor exerts an inhibitory role. These findings confirm the influence of miR-361-5p on CAS from the aspect of pathological mechanism. Consistently, The role of miR-361-5p in VSMC has been reported several times [31]. During the progression of pulmonary arterial hypertension (PAH), miR-361-5p can promote pulmonary artery smooth muscle cell viability through activating JAK2/STAT3 pathway [31]. In atherosclerosis, miR-361-5p is demonstrated to promote VSMC proliferation and inhibit cell apoptosis [16, 32]. All the evidence confirms the promoting affects of miR-361-5p on the proliferation and migration of VSMCs.

Studies have shown that miRNA exerts its biological function by participating in regulating the translation process of its downstream genes [33]. In the current study, tissue inhibitor of matrix metalloproteinase 4 (TIMP4) was prodected to be a candidate gene of miR-361-5p via bioinformatic analysis, and the target relationship was confirmed via luciferase reporter assay in VSMCs. Clinically, a negative association has been detected between plasma TIMP4 levels and the carotid intima-media thickness (CIMT) in the young people [34]. And the absence of TIMP4 is associated with atherosclerotic plaque deposition [35]. A study about cardiovascular disease has demonstrated that TIMP4 deficient mice are more susceptible to myocardial infarction, a high mortality rate induced by myocardial infarction is detected [36]. The role of TIMP4 in VSMCs has also been widely reported [37]. In a study of carotid intima formation in rats, overexpression of TIMP4 inhibited the migration of VSMCs and induce cell apoptosis [38]. In the current study, the rescue cell experiments determined that TIMP4 knockdown counteracts the role of miR-361-5p in VSMCs proliferation and migration. The conclusion was made that miR-361-5p might promoted VSMCs proliferation and migration via targeting TIMP4.

## Conclusions

In conclusion, upregulation of miR-361-5p is identified to be a promising biomarker for CAS diagnosis, and it is related to the CAS degree. In cell experiments, miR-361-5p can promote VSMCs proliferation and migration via targeting TIMP4. miR-361-5p is an independent risk factor for CAS, and can be used as a potential target for early diagnosis and treatment of CAS.

## Data Availability

The datasets used and/or analysed during the current study are available from the corresponding author on reasonable request.
